# Association of *CHRDL1* Mutations and Variants with X-linked Megalocornea, Neuhäuser Syndrome and Central Corneal Thickness

**DOI:** 10.1371/journal.pone.0104163

**Published:** 2014-08-05

**Authors:** Alice E. Davidson, Sek-Shir Cheong, Pirro G. Hysi, Cristina Venturini, Vincent Plagnol, Jonathan B. Ruddle, Hala Ali, Nicole Carnt, Jessica C. Gardner, Hala Hassan, Else Gade, Lisa Kearns, Anne Marie Jelsig, Marie Restori, Tom R. Webb, David Laws, Michael Cosgrove, Jens M. Hertz, Isabelle Russell-Eggitt, Daniela T. Pilz, Christopher J. Hammond, Stephen J. Tuft, Alison J. Hardcastle

**Affiliations:** 1 UCL Institute of Ophthalmology, London, United Kingdom; 2 Department of Twin Research and Genetic Epidemiology, King's College London, St. Thomas' Hospital, London, United Kingdom; 3 UCL Genetics Institute, London, United Kingdom; 4 Department of Ophthalmology, Centre for Eye Research, University of Melbourne, Melbourne, Victoria, Australia; 5 Moorfields Eye Hospital, London, United Kingdom; 6 Department of Ophthalmology, Odense University Hospital, Odense, Denmark; 7 Department of Clinical Genetics, Odense University Hospital, Odense, Denmark; 8 Department of Ophthalmology, Singleton Hospital, Swansea, United Kingdom; 9 Department of Women and Child Health, Singleton Hospital, Swansea, United Kingdom; 10 Great Ormond Street Hospital for Children, London, United Kingdom; 11 Institute of Medical Genetics, University Hospital of Wales, Cardiff, United Kingdom; University of Iowa, United States of America

## Abstract

We describe novel *CHRDL1* mutations in ten families with X-linked megalocornea (MGC1). Our mutation-positive cohort enabled us to establish ultrasonography as a reliable clinical diagnostic tool to distinguish between MGC1 and primary congenital glaucoma (PCG). Megalocornea is also a feature of Neuhäuser or megalocornea-mental retardation (MMR) syndrome, a rare condition of unknown etiology. In a male patient diagnosed with MMR, we performed targeted and whole exome sequencing (WES) and identified a novel missense mutation in *CHRDL1* that accounts for his MGC1 phenotype but not his non-ocular features. This finding suggests that MMR syndrome, in some cases, may be di- or multigenic. MGC1 patients have reduced central corneal thickness (CCT); however no X-linked loci have been associated with CCT, possibly because the majority of genome-wide association studies (GWAS) overlook the X-chromosome. We therefore explored whether variants on the X-chromosome are associated with CCT. We found rs149956316, in intron 6 of *CHRDL1*, to be the most significantly associated single nucleotide polymorphism (SNP) (p = 6.81×10^−6^) on the X-chromosome. However, this association was not replicated in a smaller subset of whole genome sequenced samples. This study highlights the importance of including X-chromosome SNP data in GWAS to identify potential loci associated with quantitative traits or disease risk.

## Introduction

X-linked megalocornea (MGC1; MIM 309300) is an inherited congenital disorder, characterised by bilateral enlarged corneas with a horizontal diameter of ≥13 mm (measured after the age of two years) and reduced central corneal thickness in the absence of raised intraocular pressure (IOP) [1]. Adult-onset cataract typically develops between 30–50 years [2]. Other secondary changes include mosaic corneal degeneration (shagreen), corneal arcus juvenilis, lens dislocation and mild iris atrophy with pigment dispersion [3]. The condition was genetically linked to the long arm of the X-chromosome over twenty years ago (Xq12-q26; MGC1) [2], [4] but the underlying genetic cause, mutations in *CHRDL1* (MIM 300350), has only recently been discovered [3]. *CHRDL1* encodes ventroptin (or neuralin-1, neurogenesin-1, chordin-like 1), a secreted bone morphogenetic protein (BMP) antagonist [5]. To date, presumed loss-of-function mutations in *CHRDL1* have been described in eight unrelated families affected with MGC1 [3], [6].

Distinguishing megalocornea from primary congenital glaucoma (PCG) in infants is clinically challenging due to overlapping phenotypic features. PCG is a genetically heterogeneous condition, often associated with a broad spectrum of additional phenotypes [7]. PCG is characterised by high IOP and enlarged globe size (buphthalmos) resulting from an obstruction of aqueous outflow from the anterior segment. Late detection and delayed treatment lead to irreversible and progressive damage to the optic nerve and permanent visual loss can occur [8]. Genetic analysis of the condition has resulted in identification of three PCG loci on chromosomes 2p22 (GLC3A, MIM 613086) [9], 1p36 (GLC3B, MIM 600975) [10] and 14q24.3-q31.1 (GLC3C, GLC3D, MIM 613085, MIM 613086) [11]. Recessive mutations in *CYP1B1* (GLC3A) are the most common cause, especially in populations where consanguinity is common, such as the Saudi population [12]. Variants in *MYOC* have also been associated with PCG either independently or in association with *CYP1B1* [13]. However, variants in this gene are more commonly associated with juvenile and adult-onset primary open angle glaucoma (GLC1A, MIM 137750) [14]. Recessive mutations in *LTBP2* (GLC3D, MIM 613086) have been identified as a cause of early-onset glaucoma secondary to congenital megalocornea in multiple consanguineous families [15]–[17]. Notably, in the eight reported unrelated MGC1 families with *CHRDL1* mutations, none of the affected males (ranging from 8–72 years of age) had raised IOP, glaucoma or significant visual loss [3], [6].

Megalocornea is a defining feature of megalocornea-mental retardation (MMR) syndrome [MIM 249310], also known as Neuhäuser syndrome [18]. The condition is rare and characterised by megalocornea in addition to neurological symptoms including intellectual disability (ID), hypotonia and seizures [1], [18], [19]. Syndromic facial features are also described and include a prominent forehead, broad nasal root, epicanthus, large low set ears, long upper lip and anteverted nostrils [1]. The genetic cause of MMR syndrome is currently unknown. The majority of reported cases are consistent with an autosomal recessive mode of inheritance or a *de novo* cause, and the phenotypic heterogeneity associated with cases reported to date suggests that the condition may be genetically heterogeneous [19].

Reduced central corneal thickness (CCT) is a common feature of all MGC1 patients with a *CHRDL1* mutation [3], [6]. In the general population, CCT is known to be a highly heritable, normally distributed quantitative trait, and is the focus of many genome-wide association studies (GWAS) because it is known to be a major risk factor and endophenotype for open-angle glaucoma, one of the leading causes of irreversible blindness worldwide [20]–[25]. The X-chromosome is commonly excluded from such GWAS analysis [26] and given that reduced CCT is a key feature of MGC1, here we tested whether any common variants on the X-chromosome influence CCT in the general population.

## Materials and Methods

### Study subjects and clinical examination

All investigations were conducted in accordance with the principles of the Declaration of Helsinki. The study was approved by the following local ethics committees; Moorfields and Whittington Hospital UK, The Royal Victorian Eye and Ear Hospital Australia, Odense University Hospital Denmark and University Hospital of Wales UK. Informed written consent, including permission to publish photographs, was obtained from all participating individuals or parental guardians on behalf of the minors enrolled in this study. Blood samples were donated and genomic DNA was extracted from peripheral blood lymphocytes using conventional methodologies. Patients and their relatives were clinically assessed by experienced ophthalmologists. Standard evaluation consisted of detailed ophthalmic examination and the additional measurement of the axial length of the eye and imaging of the anterior segment of the eye performed with ocular coherence tomography (OCT; Visante, Carl Zeiss Meditec), b-scan ultrasonography, and optical interferometry (IOLMaster, Carl Zeiss Meditec). Proband of Family K was clinically assessed by a geneticist (DTP), a paediatric ophthalmologist (DL) and a paediatrician (MG). He had standard MRI with a 1.5 Tesla scanner.

### Sanger sequencing

*CHRDL1* was directly Sanger sequenced from PCR amplimers as previously described [3]. Segregation analyses of disease-associated variants in *CHRDL1* were tested in additional family members, where available, by direct sequencing of the specific exons carrying the mutation. The extent of the deletions encompassing *CHRDL1* was refined by targeted proximal and distal PCRs amplifying the *CHRDL1* flanking genes *RGAG1* and *PAK3* (Families I and J). Exon 4 of the proximal gene *RGAG1* was amplified with primers F: 5′-AAGGGTGAAGGCAACAAGG-3′ and R: 5′-GCAAAGTGTCTTGATCTGCTAAG-3′, and exon 1 of the distal gene *PAK3* was amplified with primers F: 5′-AGCAGAGAAGGGCTAGGGAG-3′ and R: 5′-GTCTAGGG\TTTGACCAAGCG-3′. To confirm the deletion identified in Family H, a long range PCR was performed using BIO-X-ACT Long DNA Polymerase (Bioline) according to the manufacturer's recommendations with primers F: 5′-TGAAGCCTAGAGATGCAAAGTG-3′ in intron 4 and R: 5′-GATGGCCACAGCTCAGTCTA-3′ in intron 5. To determine the breakpoint positions, the resulting long range PCR product was directly Sanger sequenced. *CHRDL1* cDNA is numbered in accordance with Ensembl transcript ID ENST00000372042, with +1 corresponding to the A of the ATG translation initiation codon in the reference sequences.

### Whole Exome Sequencing (WES)

WES was performed using Illumina TruSeq exome enrichment (v3) and a HiSeq2000 sequencer (Illumina). Reads were aligned to the hg19 human reference sequence using Novoalign (Novocraft, www.novocraft.com) version 2.05. The ANNOVAR tool (OpenBioinformatics) was used to annotate SNPs and small insertions/deletions. Filtering was performed to identify variants with a minor allele frequency (MAF)≤0.01 in 1000 Genomes Project (www.1000genomes.org/), 6500 NHLBI Exome Sequencing Project (http://evs.gs.washington.edu/EVS/), and our internal control dataset consisting of 304 exomes. Variants were then prioritized based on their effects on the protein function (frameshift, non-synonymous, splice site, and synonymous). WES analysis was also extended to include 5′UTR and 3′UTR variants, where covered. Variants were then cross-referenced with genes that have been reported to be associated with ID and/or hypotonia and/or seizures and/or epilepsy. The table of candidate genes was generated using the KEGG disease database (www.genome.jp/kegg/) and with reference to the report by Piton *et al.* [27]. All rare variants identified by WES in Individual III:4 (Family K) in genes associated with the neurological phenotypes were verified by PCR amplification and Sanger sequencing using standard methodologies (primer sequences are available on request). Segregation of the rare variants was tested in the proband's mother (Individual II:2) by the same method (Table S1 in File S1).

### *In silico* analysis

Non-synonymous missense variants identified were scored for likely pathogenicity using SIFT (http://sift.jcvi.org/), PolyPhen2 (http://genetics.bwh.harvard.edu/pph2/) and Blosum62.

### Association study

To determine whether any common variants on the X-chromosome influence CCT in the general population, we analysed SNP array genotyping data from the TwinsUK cohort [28]. Subjects were genotyped in two different batches of approximately the same size, using two genotyping platforms from Illumina: 300K Duo and HumanHap610-Quad arrays. CCT was measured in this cohort using ultrasound pachymetry and recorded for both eyes. Measurements were performed using a DGH Technology (model 500). Data collected from 1,957 female Caucasian participants were analysed. Whole genome imputation of the genotypes (2,729 SNPs) was performed using 1000G haplotypes with the IMPUTE v2 software. Stringent quality control (QC) measures were implemented prior the imputation, including minimum genotyping success rate (>95%) and Hardy-Weinberg equilibrium (P>10^−6^). Regression analysis for association was performed using mach2qtl and the results were adjusted for age effects and family relatedness among the samples.

We checked whether this association could be replicated in a subset of directly whole genome sequenced individuals, made available through the UK10K project (http://www.uk10k.org/). A total of 760 female participants with both sequencing and phenotyping data were analysed. A regression model for association was fitted using PLINK (http://pngu.mgh.harvard.edu/~purcell/plink/) using as predictor each single variant and as outcome CCT adjusted for age effects.

## Results

### MGC1 families

**Figure 1:**
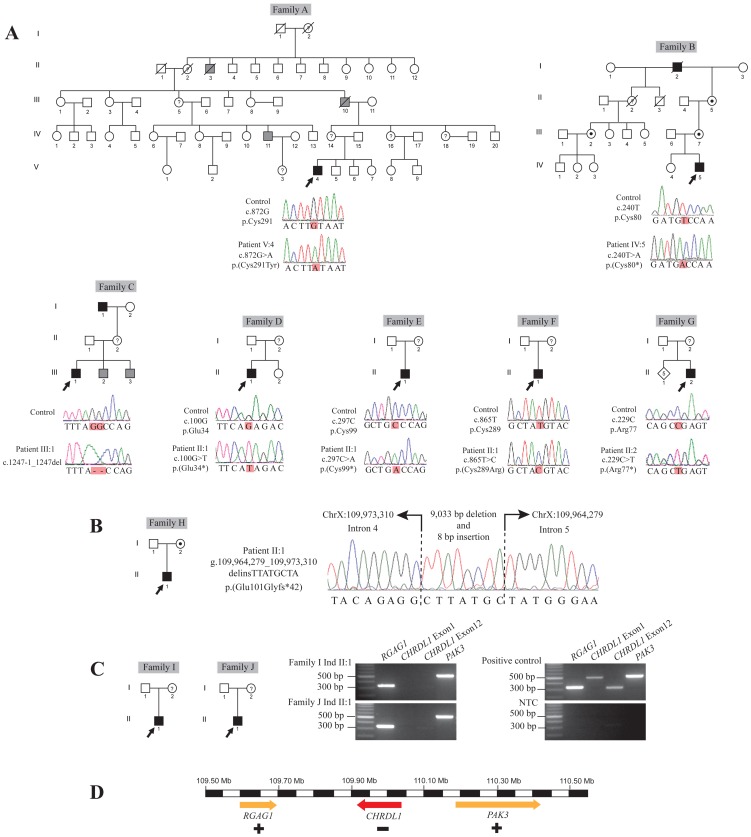
X-linked Megalocornea Families A–J. (A) Pedigree of Families A–G. Black shaded squares denote clinically and genetically confirmed affected males; grey shaded squares denote clinically diagnosed affected males but DNA samples were not available for testing; dotted circles denote genetically confirmed carrier females; ? = presumed carrier females but DNA samples were not available for testing; clear squares and circles denote unaffected individuals. Arrowhead indicates proband in each family. Control sequence electropherogram is shown above patient sequence. (B) Pedigree of Family H and sequence electropherogram showing a 9,033 bp deletion encompassing *CHRDL1* exon 5 and 8 bp insertion. (C) Pedigree of Families I and J. Deletion of the entire *CHRDL1* gene (exon 1 to exon 12) in the proband is shown in agarose gel images. The flanking genes, *RGAG1* and *PAK3* are present. NTC, non-template control (D) Schematic representation of presence or absence of the *CHRDL1* gene and flanking genes *RGAG1* and *PAK3* (Ensembl nomenclature hg19 genome build) in Families I and J.

We ascertained ten new families (A–J) with a diagnosis of MGC1 (Figure 1; Table 1), the largest cohort reported to date. Ophthalmic features of all patients are described in Table 1. Consistent with our previous findings [3], all patients were identified as having large horizontal corneal diameters (ranging from 13–16 mm, white to white) and a below average CCT, ranging from 346–475 µm with the mean CCT being 418 µm (1SD = 33 µm; number eyes measured = 30) (Table 1 and [3]). The reported mean value for CCT in normal male and female adult eyes is 535 µm (1SD = 31 µm) [29]. Deep anterior chamber depths were noted in all examined MGC1 subjects, ranging from 4.30–6.50 mm (Table 1). In the normal population of predominantly European children (mean age 6.7 years), the mean axial length in right eyes was 22.61 (+/−0.02) mm and the mean anterior chamber depth was 3.34 (+/−0.01) mm [30]. In an older population of white adults (>65 years) mean axial length and anterior chamber depth were found to be 23.69 (+/−1.16) mm and 3.11 (+/−0.37) mm, respectively [31]. IOPs for all MGC1 subjects were determined to be within normal limits. Cataract surgery had been performed in all three individuals above the age of 40 years. Additional secondary features of the condition including shagreen, arcus juvenilis, and mild iris atrophy with pigment dispersion were also age-related (Table 1; Figure S1 in File S1).

**Table 1:** Ocular phenotype: X-linked megalocornea patients.

	Family A		Family B		Family C		Family D		Family E		Family F		Family G		Family H		Family I		Family J		Family K	
	V:4		IV:5		III:1		II:1		II:1		II:1		II:2		II:1		II:1		II:1		III:4	
	R	L	R	L	R	L	R	L	R	L	R	L	R	L	R	L	R	L	R	L	R	L
**Maternal ethnicity**	White		White		White		White		White		White		White		Asian		White		White		White	
**Age at last follow up (years)**	44		2		18		15		58		1		2		0.5		2		72		8	
**Refraction (before surgery)**	−9.50/−1.50×70	−7.25/−1.00×180	+1,5/−0,75 cyl 15	+1,5/−0,25 cyl 149	−4.75/−1.0×180	−3.5/−2.25×170	−7.5/−3.0×20	−5.5/−3.75×5	−2.5/−1.75×25	−3.5/−2.75	+2.5/−3.00×40	+2.75/−3.75×41	+3.0/−2.25×15	+2.5/−2.0×5	−1.00	−1.00	+1.00/−0.5×20	+1.5/−0.5×160	NA		+0.25/−	0/+0.25×90
**Cataract surgery (years)**	43	43	−	−	−	−	−	−	56	42	−	−	−	−	−	−	−	−	60	60	−	−
**Retinal detachment (age)**	−	−	−	−	−	−		−	−	+(46)	−	−	−	−	−	−	−	−	+(62)	−	−	−
**Visual acuity (LogMAR)**	6/6	6/9	6/12	6/12	6/5	6/5	0.3	0.1	6/5	6/60	>6/76	>6/76	>0.8	>0.8	NA	NA	NA	NA	6/6	6/6	0.02	0.02
**Iris transillumination**	+	+	−	−	−	−	−	−	+	+	+	+	−	−	−	−	NA	NA	+	+	−	−
**Arcus juvenilis**	+	+	−	−	−	−	−	−	+	+	−	−	−	−	−	−	−	−	+	+	−	−
**Shagreen**	+	+	−	−	−	−	−	−	+	+	−	−	−	−	−	−	−	−	+	+	−	−
**Anterior chamber (AC) depth (mm)**	6.30	6.30	NA	NA	5.08	4.82	5.72	5.78	NA	6.39	4.30	4.40	4.30	4.50	NA	NA	NA	NA	NA	6.50	5.15	4.84
**HWTW (mm)**	15	15	16	15.5	16	16	15	15	15.9	15.9	15	15	15	15	13	13	14.75	14.75	14.9	14.9	14	13.5
**Axial length (AL) (mm)**	27.9	27.9	23.8	23.6	26.1	25.4	27.31	26.6	28.69	27.91	21.0	22.5	22.4	21.8	NA	NA	21.75	21.8	27.95	27.36	22.91	23.14
**Central corneal thickness (µm)**	440	427	446	NA	430	470	403	406	470	475	406	406	NA	NA	NA	NA	419	409	NA	408	N/A	N/A
**Disc (cup:disc)**	0.3	0.3	0.1	0.1	0.1	0.1	0.1	0.1	0.3	0.3	0.1	0.1	0.1	0.1	0.1	0.1	0.1	0.1	0.4	0.3	0.3	0.3
**AC/AL ratio**	0.23	0.23	NA	NA	0.19	0.19	0.21	0.22	NA	0.23	0.20	0.20	0.19	0.21	NA	NA	NA	NA	NA	0.24	0.22	0.21
**IOP (mmHg)**	10	11	11	10	12	12	12	12	18	17	10	10	8	9	12	12	12	13	16	16	10	10

The following abbreviations and symbols have been used: NA = not available; HWTW = cornea horizontal diameter (white to white); IOP = intraocular pressure.

### Novel *CHRDL1* mutations causing MGC1

For each affected proband (Families A–J), *CHRDL1* exons and intron-exon boundaries were screened by bi-directional Sanger sequencing. In total, ten *CHRDL1* mutations were identified in each of the ten respective families investigated (Figure 1; Table 2).

**Table 2:** Summary of *CHRDL1* mutations identified.

Family Number	Nucleotide change	Protein change	Polyphen 2 (human variation score 0–1)	SIFT (tolerance index 0–1)	Blosum 62 score (−4 to 11)	ESP Total alleles	Reported
**A**	**c.872G>A**	**p.(Cys291Tyr)**	**PRD (0.998)**	**TOLERATED (0.1)**	**−2**	**0/10,562**	**This study**
**B**	**c.240T>A**	p.(Cys80*)	**NA**	**NA**	**NA**	**0/10,562**	**This study**
**C**	**c.1247-1_1247del**	**Unknown**	**NA**	**NA**	**NA**	**0/10,562**	**This study**
**D**	**c.100G>T**	p.(Glu34*)	**NA**	**NA**	**NA**	**0/10,562**	**This study**
**E**	**c.297C>A**	p.(Cys99*)	**NA**	**NA**	**NA**	**0/10,562**	**This study**
**F**	**c.865T>C**	**p.(Cys289Arg)**	**PRD (0.998)**	**DAMAGING (0)**	**−3**	**0/10,562**	**This study**
**G**	**c.229C>T**	p.(Arg77*)	**NA**	**NA**	**NA**	**0/10,5622**	**This study**
**H**	**g.109964279_109973310delinsTTATGCTA**	p.(Glu101Glyfs*42)	**NA**	**NA**	**NA**	**0/10,562**	**This study**
**I**	**Whole gene deletion**	**No protein**	**NA**	**NA**	**NA**	**NA**	**This study**
**J**	**Whole gene deletion**	**No protein**	**NA**	**NA**	**NA**	**NA**	**This study**
**K**	**c.464G>A**	**p.(Cys155Tyr)**	**POS (0.796)**	**TOLERATED (0.4)**	**−2**	**0/10,562**	**This study**
1	Segmental deletion	Truncated protein product	NA	NA	NA	NA	[3]
2	c.102_103del	p.(Glu34Aspfs*14)	NA	NA	NA	0/10,562	[3]
3	c.782G>T	p.(Cys261Phe)	PRD (0.998)	DAMAGING (0)	−2	0/10,562	[3]
4	c.301+2T>G	Unknown	NA	NA	NA	0/10,562	[3]
5	c.872del	p.(Cys291Leufs*25)	NA	NA	NA	0/10,562	[3]
6	c.652C>T	p.(Arg218*)	NA	NA	NA	0/10,562	[3]
7	Segmental deletion	No protein	NA	NA	NA	NA	[3]
NA	c.167del	p.(Pro56Leufs*8)	NA	NA	NA	0/10,562	[6]

*In silico* analysis of rare *CHRDL1* variants identified is presented. Polyphen 2 appraises mutations quantitatively as benign, possibly damaging (POS) or probably damaging (PRD) based on the model's false positive ratio. SIFT results are reported to be tolerant if tolerance index is ≥0.05 or intolerant if tolerance index is <0.05. Blosum62 substitution matrix score positive numbers indicate a substitution more likely to be tolerated evolutionarily and negative numbers suggest the opposite. The cDNA is numbered according to Ensembl transcript ID ENST00000372042. ESP denotes variants in the NHLBI ESP [accessed 26^th^ March 2014]. Mutations reported in this study are highlighted in bold. NA = not available.

**Figure 2:**
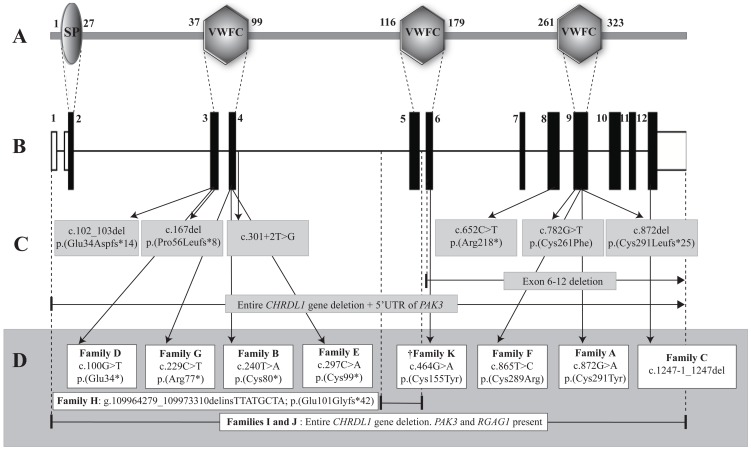
Summary of *CHRDL1* mutations. (A) Schematic of CHRDL1 protein domains. The following abbreviations are used: SP, signal peptide; VWFC, von Willebrand factor, type C domain. (B) Schematic of the *CHRDL1* gene. (C) *CHRDL1* mutations previously reported in X-linked megalocornea families [3], [6]. Frameshift, splicing, nonsense, missense, and whole gene deletion mutations were identified. (D) Novel *CHRDL1* mutations identified in MGC1 families in this study (Families A–J). The dagger (†) indicates Family K with MMR syndrome.

Affected Individual V:4 from Family A was identified as harbouring a novel missense mutation in *CHRDL1*, c.872G>A, p.(Cys291Tyr). Ventroptin has three highly conserved cysteine-rich von Willebrand factor, type C (VWFC) domains, and Cys291 is within the third domain (Figures 1A, 2D and S2 in File S1). A strong X-linked family history of megalocornea is reported in Family A, however further samples were not available for segregation analysis. Secondary changes in adults with megalocornea include an appearance similar to pigment dispersion syndrome, but without a Krukenberg spindle on the corneal endothelium. Proband (V:4) was a high myope (RE −8.00, LE −9.00), who exhibited unusually marked posterior iris bowing with atrophy, peripheral iris transillumination defects and increased trabecular meshwork pigmentation (Figure S1 in File S1). Aged 43 years he had bilateral phacodonesis with nuclear sclerotic cataracts, but normal pressures and no evidence of glaucomatous optic neuropathy.

The proband in Family B (Individual IV:5) was found to have a unique hemizygous nonsense mutation in *CHRDL1*, c.240T>A, p.(Cys80*). Segregation analysis revealed that the proband's mother (III:7), maternal grandmother (II:5) and maternal half great aunt (III:2) were all heterozygous carriers of this mutation. His sister (IV:4) was found to be wild-type for the variant. Interestingly, affected relatives of this Danish family were originally reported in the 1960s [32]. The proband's affected great maternal grandfather, Individual I:2 (Figure 1A), corresponds with Individual IV-7 in the original article with a greatly extended version of the pedigree spanning 7 generations and detailing many affected males [32].

In Family C, Individual III:I and his maternal grandfather (I:1) were found to have a novel 2 bp deletion affecting the splice acceptor site of *CHRDL1* exon 12; c.1247-1_1247del. This mutation is predicted to cause aberrant pre-mRNA splicing of the *CHRDL1* transcript *in vivo*. His two younger brothers have also been diagnosed with the same condition; however their DNA samples were not available for testing (Figure 1A). The probands in Family D (Individual II:1) and Family E (Individual II:1) were both identified to have novel hemizygous nonsense mutations in *CHRDL1*; c.100G>T, p.(Glu34*) and c.297C>A, p.(Cys99*), respectively (Figure 1A). Individual II:1 in Family F was found to have a unique missense mutation in *CHRDL1*, c.865T>C, p.(Cys289Arg) (Figure 1A). Similar to other missense mutations (Family A and [3]), the affected residue is positioned within a highly conserved cysteine-rich VWFC (Figure 2 and S2 in File S1). A novel nonsense mutation in *CHRDL1*, c.229C>T, p.(Arg77*) was identified in Individual II:2 in Family G. The proband's maternal uncle was reported to be affected with megalocornea, however, no DNA was available for testing.

The proband in Family H (Individual II:1) was initially identified as harbouring a deletion of *CHRDL1* exon 5 by PCR. In order to confirm the deletion and define the deletion breakpoints, a fragment of DNA spanning intron 4 to intron 5 was amplified and sequenced, revealing a 9,033 bp deletion and 8 bp insertion; g.109964279_109973310delinsTTATGCTA. The deletion encompassed exon 5, and partial sequence from intron 4 and intron 5, and is predicted to result in a frameshift and the introduction of premature termination codon, p.(Glu101Glyfs*42) (Figure 1B). Segregation analysis of the deletion confirmed that the proband's mother is a carrier and the father is wild-type.

Larger deletions encompassing the entire *CHRDL1* gene (12 exons) were identified in the affected males in Families I and J, by PCR. Successful amplification of the closest exons of neighbouring genes, *RGAG1* and *PAK3*, demonstrated in both instances that the deletions identified do not encompass any additional currently annotated genes (Figure 1C and D).

### Ultrasonography as a diagnostic tool for MGC1

**Figure 3:**
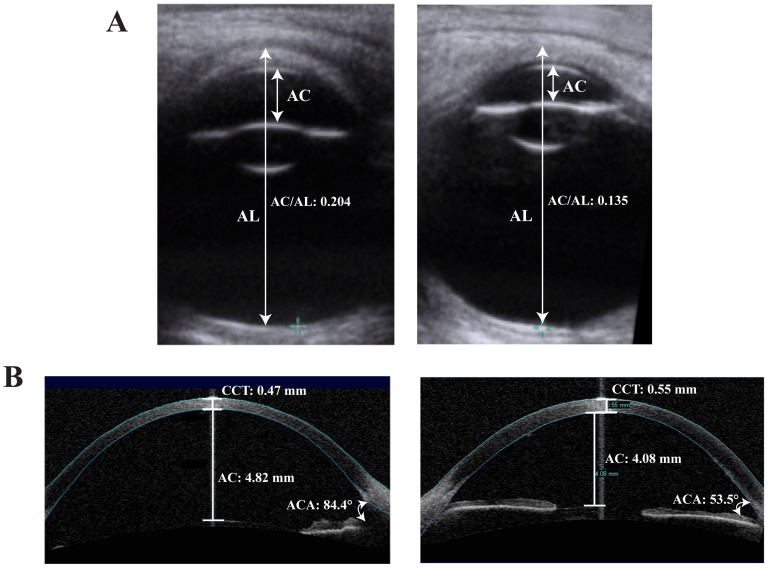
Comparison between biometry of X-linked megalocornea and congenital glaucoma. (A) AC, anterior chamber depth (back of cornea to front of lens); AL, axial length (anterior corneal surface to vitreo-retinal interface at the back of the macular). Transverse B-Mode ultrasound scans demonstrate higher ratio of the AC to AL in megalocornea, 0.204 (left) than congenital glaucoma, 0.135 (right). (B) Ocular coherence tomography (OCT) images comparing megalocornea (left) and arrested congenital glaucoma (right). Although there is enlargement of the anterior segment in both conditions, patients with megalocornea tend to have a greater anterior chamber (AC) depth, lower central corneal thickness (CCT), and deeper anterior chamber angles (ACA) than congenital glaucoma patients.

Our molecular diagnosis of MGC1 patients, here and previously [3], has allowed us to define the key phenotypic characteristics of MGC1 (Table 1) and to differentiate them from PCG (Figure 3). Patients with MGC1 have a large corneal diameter and thin cornea, but without corneal oedema or evidence of breaks in Descemet's layer. The depth of the anterior chamber in MGC1 is also typically significantly greater than in patients with PCG. Ultrasonography is a reliable clinical diagnostic tool to distinguish the two conditions and, if on ultrasonography, the ratio of the anterior chamber depth to the total axial length is ≥0.19 then a diagnosis of MGC1 is extremely likely, unless there is gross coexisting axial myopia (>36 mm) (Figure 3; Table 1).

### MMR syndrome

**Figure 4:**
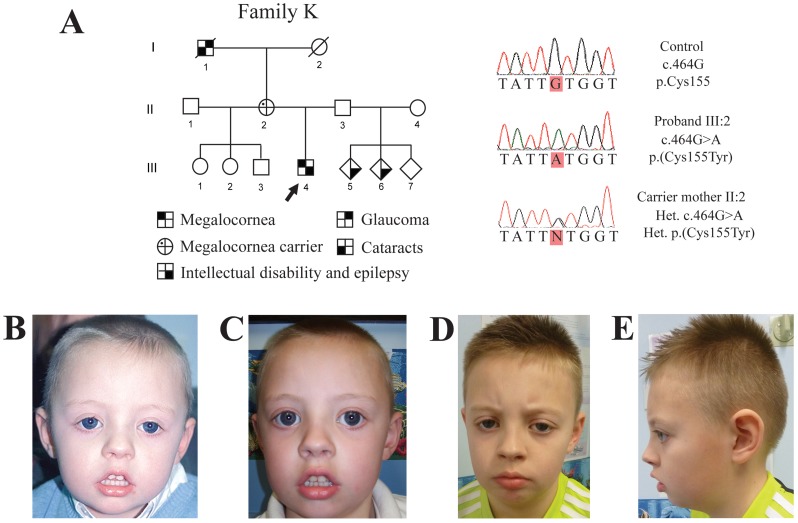
Novel *CHRDL1* missense mutation in a patient diagnosed with MMR. (A) Pedigree of Family K with megalocornea-mental retardation (MMR) syndrome. Squares, males; circles, females; diamonds, unknown gender; shaded, affected; dotted, carrier; clear, unaffected. Arrowhead indicates proband. Sequence electropherograms show the *CHRDL1* missense mutation c.464G>A; p.(Cys155Tyr), which segregates in Family K. (B–C) The proband at ages 3, and 6 years, respectively, presented with bilateral megalocorneae, broad forehead, bilateral epicanthic folds, a tented upper lip, and downturned corners of the mouth. (D–E) Frontal and sides of the proband at age of 10 years.

The male proband in Family K is the only child of his parents, who are unrelated. Pregnancy was complicated by 7 days of vaginal bleeding at 8 weeks gestation, and persistent abdominal pain from 17 weeks of pregnancy. Due to the abdominal pain, labour was induced at 39 weeks gestation. Delivery was normal and he weighed 3.917 kg (91^st^ centile). In the first week of life, he had some choking episodes, which were thought to be due to gastro-oesophageal reflux. Thereafter, feeding and weight gain were satisfactory. He had an apnoeic attack at the age of 7 weeks. At the age of 3 months, he developed epilepsy and was started on Sodium Valproate. By 6 months of age, there were concerns about his psychomotor development. He was found to have global developmental delay; he only walked at the age of 20 months and he required speech therapy. Bilateral megalocorneae were diagnosed in infancy. Physical features at the age of 3 years included a broad forehead, bilateral megalocorneae, bilateral epicanthic folds, a tented upper lip and downturned corners of the mouth (Figure 4B). His occipital frontal circumference (OFC) was on the 25^th^ centile. Magnetic resonance imaging (MRI) of his brain and karyotying were reported as normal. He was also very hypotonic initially, but this has improved. On the basis of his medical history and physical features, a diagnosis of MMR syndrome was made. At the age of 10 years, he has moderate intellectual disability (ID) and a diagnosis of autistic spectrum disorder. His antiepileptic medication had been discontinued at the age of 9 years, and he has had no further seizures. He has episodes of challenging behaviour. Growth parameters were 25^th^–50^th^ centile for height, 91^st^–98^th^ centile for weight, and his OFC was between the 9^th^ and 25^th^ centile.

Neither of the proband's parents have a history of megalocornea, ID or epilepsy. There is no paternal contact. His mother had laser iridotomies at the age of 47 years for bilateral, narrow anterior chamber drainage angles. He has 2 maternal half-sisters and a maternal half-brother, who are healthy. His maternal grandparents are both deceased; his maternal grandfather is reported to have had “large eyes”, cataracts and glaucoma. He also has 3 paternal half-siblings. Two of them are said to have epilepsy and ID, although this may be due to infantile meningitis in one of the children.

### Identification of a *CHRDL1* missense mutation in a male patient diagnosed with MMR syndrome

Given the rarity of MMR syndrome and the phenotypic variability reported in the literature [19], we hypothesised that the condition may not be genetically homogeneous. Since megalocornea is a primary feature of MMR syndrome, we considered that in this affected male (Individual III:4 Family K, Figure 4), the megalocornea phenotype may be due to a mutation in *CHRDL1*. Sanger sequencing of *CHRDL1* revealed a unique hemizygous missense mutation, c.464G>A, p.(Cys155Tyr) (Table 2) in the proband within a conserved cysteine-rich VWFC domain. His unaffected mother was identified to be a heterozygous carrier (Figure 4A). Despite mutations in *CHRDL1* being previously associated with abnormal brain morphology [3], it was unlikely that his extraocular features were caused by the *CHRDL1* mutation because no other patients with a mutation in *CHRDL1* present with developmental delay or any other of the extraocular phenotypes associated with MMR syndrome [3].

### WES analysis of genes previously associated with ID, epilepsy, seizures and hypotonia

Next, we considered the possibility that the extraocular phenotypes may be due to a mutation in an X-linked intellectual disability (XLID) gene in linkage disequilibrium (LD) with the *CHRDL1* mutation, or an autosomal ID gene. We therefore performed WES with the aim of determining if any additional genetic variants could be contributing to his extraocular features.

Overall 21,210 exonic and 61,579 total genomic sequence alterations were identified by WES, with respect to the reference sequence. As causal variants are hypothesised to be rare, we filtered the dataset for rare variants with a MAF≤0.01 in 1000 Genomes Project, 6500 NHLBI Exome Sequencing Project, and our internal control data set consisting of 304 exomes. WES further confirmed the *CHRDL1* mutation. We created a database of genes that have been associated with ID and/or hypotonia and/or seizures and/or epilepsy, and cross-referenced the filtered rare variants dataset against this database. Using this approach, we identified ten rare variants of unknown significance in genes associated with these neurological phenotypes including four heterozygous missense variants, two heterozygous synonymous variants and four variants present within non-protein coding regions (5′ and 3′ UTR regions) (Table S1 in File S1). Each of the ten variants was validated by direct Sanger sequencing in the proband and his unaffected mother; paternal DNA was not available for testing. Two rare autosomal variants in *SEMA3E* (MIM 608166) and *DDB1* (MIM 600045) were identified in the proband and his unaffected mother in the heterozygous state, suggesting that they are unlikely to be causative of the extraocular phenotypes observed (Table S1 in File S1). Five of the remaining eight heterozygous variants were identified in genes associated with autosomal recessive forms of disease (*ERCC8, UROC1, ADCK3, ERCC6, and ERCC3*) suggesting that, in isolation, each of these rare variants would also be unlikely to be causative.

A unique heterozygous variant (g.161326951 A>C) was identified in the 3′UTR of *GABRA1* (MIM 137160, Table S1 in File S1). Non-synonymous heterozygous variants in *GABRA1* have been associated with juvenile myoclonic epilepsy [33]. However, considering the rarity of critical regulatory elements in 3′UTRs, a mutation in this region is usually tolerated. It is therefore unlikely that this variant would be causative. The proband's mother (Individual II:2) was wild-type for this variant, so either the proband inherited the variant from his father (Individual II:3), or it is a *de novo* change. The proband's father has no reported history of epilepsy.

A rare heterozygous missense variant, p.(Thr1016Asn), absent in the proband's mother, was identified in *DIP2B* (MIM 611379, Table S1 in File S1). An expansion of a CGG repeat in the 5′UTR of *DIP2B* has been reported in two patients with ID (FRA12A) [34], however no missense variants have been associated with disease.

A unique hemizygous 1 bp deletion in the 5′UTR region of *CUL4B* (MIM 300304) was identified in the proband (Table S1 in File S1). Interestingly, a 2 Kb non-coding deletion that disrupts the 5′UTR of *CUL4B* has previously been identified to co-segregate with ID in a single family, resulting in loss of *CUL4B* expression, suggestive of a pathogenic effect [35]. Mutations in this gene in several families with XLID were initially reported in 2007 [36], [37]. Interfamilial and intrafamilial phenotypic variability was noted with regard to several features, including the degree of ID and the growth parameters (height and OFC). Additional publications further confirmed the association with moderate to severe ID and marked speech delay in all patients. Challenging behaviour, seizures, and a prominent lower lip were also common, as seen in our patient [38], [39].

Notably, *CUL4B* is situated approximately 900 Kb downstream of *CHRDL1*. Since the proband's mother is also a carrier of this variant, it is likely that the rare variants identified in *CUL4B* and *CHRDL1* are in LD. This suggests that MMR in this patient may be a digenic X-linked trait. However, the proband's deceased maternal grandfather reportedly had ‘large eyes’ in the absence of extraocular features. This implies that he may have had MGC1 and the *CUL4B* variant could have occurred as a *de novo* event in the proband's mother. However, the extended family pedigree indicates a possible paternal transmission of ID and epilepsy in the proband and his affected half-siblings, which would exclude an X-linked gene.

Unfortunately, we were not able to test these hypotheses due to lack of familial DNA samples, so the potential pathogenicity of the *CUL4B* and other variants identified, and therefore the genetic cause of extraocular phenotypes in the proband, remains unresolved.

### Association study

CCT is a normally distributed (Figure S3 in File S1), highly heritable quantitative trait in the human population [40]. Despite many GWAS investigating CCT-associated loci, the X-chromosome has been largely ignored. We therefore wanted to determine whether common variants on the X-chromosome in the general population are associated with CCT. To test this hypothesis we examined SNP array genotyping data for 1,957 female participants of British descent from the TwinsUK cohort. Their mean CCT was 545.24 µm (±40.03 µm). The most significant CCT-associated SNP on the entire X-chromosome was rs149956316 (p = 6.81×10^−6^) (Figure S4 in File S1). This SNP is located within intron 6 of *CHRDL1*, and the positive effect allele (A) has an MAF of 0.017 in the TwinsUK dataset (0.008 in the 1000 Genomes dataset). To assess potential functionality of the rs149956316 variant, we compared the effect of the reference and variant allele using pre-mRNA splicing prediction programs including Human Splicing Finder (HSF) [41], NNSPLICE [42], NetGene2 [43] and SpliceAid [44], but there were no predicted motif alterations or alternative splicing activity.

Given the known limitations of genomic imputation for variants with low MAF, we attempted a replication of these results in a smaller subset of 760 individuals from the TwinsUK cohort, for which both CCT and genome sequence data were available. We did not observe a significant effect in this smaller sample and were unable to replicate the results from the imputation. The current conflicting data makes it impossible to determine if this non-validation is the result of insufficient power from the limited number of samples available, accumulation of potentially influential imputation mistakes, or if the initial imputed observation was purely a type one error.

This data initially suggested that the *CHRDL1* locus may be a quantitative trait locus for CCT, however, given the uncertainties during the replication in directly acquired genotypes (as opposed to imputed ones), further validation is warranted before the *CHRDL1* locus can be substantiated as influencing CCT in the general population.

## Discussion

All MGC1 families ascertained in this study were found to have unique *CHRDL1* mutations including nonsense mutations p.(Glu34*), p.(Arg77*), p.(Cys80*) and p.(Cys99*), missense mutations p.(Cys289Arg) and p.(Cys291Tyr), a frameshift mutation p.(Glu101Glyfs*42), a splice site mutation (c.1247-1_1247del) and deletions encompassing the entire *CHRDL1* transcript. To date, all patients recruited to our study with a diagnosis of MGC1 were found to have a mutation in *CHRDL1* [3].

Members of the transforming growth factor beta (TGF-β) superfamily, including BMPs, are known to play important roles in ocular development [45], [46]. The concentration of active BMP ligands is tightly regulated by a collection of secreted proteins, including the chordin family, which act as BMP antagonists. The chordin proteins interact with BMPs via the conserved cysteine-rich VWFC domains [47]. *CHRDL1* encodes ventroptin, which contains three VWFC domains. Consistent with our previous findings [3], all missense mutations identified to date are located at highly conserved cysteine residues within these domains, which are predicted to abrogate interaction with BMPs.

There is an important clinical need to differentially diagnose MGC1 from PCG. In our molecularly diagnosed cohort of MGC1 patients, we have been able to use ocular imaging define the key clinical characteristics that assist in differentiating MGC1 from PCG (Figure 3). Since we have shown that *CHRDL1* mutations do not lead to increased IOP or glaucoma (Table 1) [3], the repeated examinations that are required to monitor IOP (under anaesthesia) for PCG patients are not required for MGC1 patients. A molecular genetic test for *CHRDL1* can confirm a diagnosis of MGC1 and has important implications for clinical care.

As described, megalocornea is also a key pathognomonic feature of MMR syndrome. Intriguingly, we identified another novel missense mutation in *CHRDL1*, c.464G>A; p.(Cys155Tyr) in a male patient with a diagnosis consistent with MMR syndrome. This *CHRDL1* mutation accounts for the ocular phenotype, and although the genetic cause of the extraocular phenotypes in this individual currently remains unexplained, this is the first report revealing a causative mutation in an MMR case. Recruitment of more MMR patients and a more comprehensive delineation of MMR syndrome are essential in order to investigate the underlying genetic cause(s) of this poorly defined syndrome. Our findings demonstrate that *CHRDL1* should be considered and screened in males diagnosed with MMR syndrome. Further WES and copy number variant analysis of male and female subjects will help to define the contribution of X-linked and autosomal genes to this syndrome.

We observe that an X-linked locus may be associated with CCT. In addition to MGC1, corneal thinning is associated with rare connective tissue disorders such as Brittle cornea syndrome (BCS; MIM 229200) [48] and Ehlers-Danlos syndrome (EDS; MIM 130000) [49]. Notably, common variants in genes associated with certain forms of these rare conditions, *ZNF469* (BCS; MIM 612078) and *COL5A1* (EDS; MIM 120215), have been shown by numerous GWAS to be associated with CCT in the general population [20]–[25]. The potential association of a *CHRDL1* SNP (rs149956316) with CCT mirrors these findings, and may have not been detected previously because non-autosomal data have largely been excluded from the majority of CCT GWAS studies [26]. Currently, the majority of GWAS focus upon analysis of autosomal variants only, as analysis of sex chromosomes for GWAS remains technically challenging [26], [50]. However, given the lack of replication in this study, it will be important to test for association of X-linked loci, and the *CHRDL1* locus, with CCT in male cohorts, because it is possible that phenotypic effects of X-linked loci (including the *CHRDL1* locus) may be masked in females, who have two copies of the X-chromosome. In males, in the hemizygous state, we hypothesise that a greater effect on corneal phenotypes may be observed. Our study highlights the potential importance of analysing X-chromosome SNP data in GWAS to identify loci associated with quantitative traits or disease risk.

## Supporting Information

File S1 **File includes Figures S1–S4 and Table S1.**
(DOC)

## References

[1] Meire FM (1994) Megalocornea. Clinical and genetic aspects. Documenta ophthalmologica Advances in ophthalmology 87: 1–121. 7835180 10.1007/BF01676641

[2] Meire FM, Bleeker-Wagemakers EM, Oehler M, Gal A, Delleman JW (1991) X-linked megalocornea. Ocular findings and linkage analysis. Ophthalmic paediatrics and genetics 12: 153–7. 1754164 10.3109/13816819109029398

[3] Webb TR, Matarin M, Gardner JC, Kelberman D, Hassan H, et al (2012) X-linked megalocornea caused by mutations in CHRDL1 identifies an essential role for ventroptin in anterior segment development. American journal of human genetics 90: 247–59. 22284829 10.1016/j.ajhg.2011.12.019 PMC3276677

[4] Chen JD, Mackey D, Fuller H, Serravalle S, Olsson J, et al (1989) X-linked megalocornea: close linkage to DXS87 and DXS94. Human genetics 83: 292–4. 2571565 10.1007/BF00285176

[5] Gao WL, Zhang SQ, Zhang H, Wan B, Yin ZS (2013) Chordin-like protein 1 promotes neuronal differentiation by inhibiting bone morphogenetic protein-4 in neural stem cells. Molecular medicine reports 7: 1143–8. 23404565 10.3892/mmr.2013.1310

[6] Han J, Young JW, Frausto RF, Isenberg SJ, Aldave AJ (2013) X-linked Megalocornea Associated with the Novel CHRDL1 Gene Mutation p.(Pro56Leu*8). Ophthalmic genetics 10.3109/13816810.2013.837187 PMC3968246 24073597

[7] Sharafieh R, Child AH, Khaw PT, Fleck B, Sarfarazi M (2013) LTBP2 gene analysis in the GLC3C-linked family and 94 CYP1B1-negative cases with primary congenital glaucoma. Ophthalmic genetics 34: 14–20. 22924778 10.3109/13816810.2012.716486

[8] Kupfer C, Kaiser-Kupfer MI (1979) Observations on the development of the anterior chamber angle with reference to the pathogenesis of congenital glaucomas. American journal of ophthalmology 88: 424–6. 484670 10.1016/0002-9394(79)90643-3

[9] Sarfarazi M, Akarsu AN, Hossain A, Turacli ME, Aktan SG, et al (1995) Assignment of a locus (GLC3A) for primary congenital glaucoma (Buphthalmos) to 2p21 and evidence for genetic heterogeneity. Genomics 30: 171–7. 8586416 10.1006/geno.1995.9888

[10] Akarsu AN, Turacli ME, Aktan SG, Barsoum-Homsy M, Chevrette L, et al (1996) A second locus (GLC3B) for primary congenital glaucoma (Buphthalmos) maps to the 1p36 region. Human molecular genetics 5: 1199–203. 8842741 10.1093/hmg/5.8.1199

[11] Stoilov IR, Sarfarazi M (2002) The Third Genetic Locus (GLC3C) for Primary Congenital Glaucoma (PCG) Maps to Chromosome 14q24.3. Invest Ophthalmol Vis Sci 2002

[12] Abu-Amero KK, Osman EA, Mousa A, Wheeler J, Whigham B, et al (2011) Screening of CYP1B1 and LTBP2 genes in Saudi families with primary congenital glaucoma: genotype-phenotype correlation. Molecular vision 17: 2911–9. 22128238 PMC3224840

[13] Kaur K, Reddy AB, Mukhopadhyay A, Mandal AK, Hasnain SE, et al (2005) Myocilin gene implicated in primary congenital glaucoma. Clinical genetics 67: 335–40. 15733270 10.1111/j.1399-0004.2005.00411.x

[14] Fingert JH, Heon E, Liebmann JM, Yamamoto T, Craig JE, et al (1999) Analysis of myocilin mutations in 1703 glaucoma patients from five different populations. Human molecular genetics 8: 899–905. 10196380 10.1093/hmg/8.5.899

[15] Ali M, McKibbin M, Booth A, Parry DA, Jain P, et al (2009) Null mutations in LTBP2 cause primary congenital glaucoma. American journal of human genetics 84: 664–71. 19361779 10.1016/j.ajhg.2009.03.017 PMC2680998

[16] Desir J, Sznajer Y, Depasse F, Roulez F, Schrooyen M, et al (2010) LTBP2 null mutations in an autosomal recessive ocular syndrome with megalocornea, spherophakia, and secondary glaucoma. European journal of human genetics: EJHG 18: 761–7. 20179738 10.1038/ejhg.2010.11 PMC2987369

[17] Narooie-Nejad M, Paylakhi SH, Shojaee S, Fazlali Z, Rezaei Kanavi M, et al (2009) Loss of function mutations in the gene encoding latent transforming growth factor beta binding protein 2, LTBP2, cause primary congenital glaucoma. Human molecular genetics 18: 3969–77. 19656777 10.1093/hmg/ddp338

[18] Neuhauser G, Kaveggia EG, France TD, Opitz JM (1975) Syndrome of mental retardation, seizures, hypotonic cerebral palsy and megalocorneae, recessively inherited. Zeitschrift fur Kinderheilkunde 120: 1–18. 1172332 10.1007/BF00443795

[19] Gutierrez-Amavizca BE, Juarez-Vazquez CI, Orozco-Castellanos R, Arnaud L, Macias-Gomez NM, et al (2013) Neuhauser syndrome: a rare association of megalocornea and mental retardation. Review of the literature and further phenotype delineation. Genet Couns 24: 185–91. 24032289

[20] Gao X, Gauderman WJ, Liu Y, Marjoram P, Torres M, et al (2013) A genome-wide association study of central corneal thickness in Latinos. Investigative ophthalmology & visual science 54: 2435–43. 23493294 10.1167/iovs.13-11692 PMC3621577

[21] Hoehn R, Zeller T, Verhoeven VJ, Grus F, Adler M, et al (2012) Population-based meta-analysis in Caucasians confirms association with COL5A1 and ZNF469 but not COL8A2 with central corneal thickness. Human genetics 131: 1783–93. 22814818 10.1007/s00439-012-1201-3

[22] Lu Y, Dimasi DP, Hysi PG, Hewitt AW, Burdon KP, et al (2010) Common genetic variants near the Brittle Cornea Syndrome locus ZNF469 influence the blinding disease risk factor central corneal thickness. PLoS genetics 6: e1000947. 20485516 10.1371/journal.pgen.1000947 PMC2869325

[23] Lu Y, Vitart V, Burdon KP, Khor CC, Bykhovskaya Y, et al (2013) Genome-wide association analyses identify multiple loci associated with central corneal thickness and keratoconus. Nature genetics 45: 155–63. 23291589 10.1038/ng.2506 PMC3720123

[24] Vitart V, Bencic G, Hayward C, Skunca Herman J, Huffman J, et al (2010) New loci associated with central cornea thickness include COL5A1, AKAP13 and AVGR8. Human molecular genetics 19: 4304–11. 20719862 10.1093/hmg/ddq349

[25] Vithana EN, Aung T, Khor CC, Cornes BK, Tay WT, et al (2011) Collagen-related genes influence the glaucoma risk factor, central corneal thickness. Human molecular genetics 20: 649–58. 21098505 10.1093/hmg/ddq511

[26] Wise AL, Gyi L, Manolio TA (2013) eXclusion: toward integrating the X chromosome in genome-wide association analyses. American journal of human genetics 92: 643–7. 23643377 10.1016/j.ajhg.2013.03.017 PMC3644627

[27] Piton A, Redin C, Mandel JL (2013) XLID-Causing Mutations and Associated Genes Challenged in Light of Data From Large-Scale Human Exome Sequencing. American journal of human genetics 10.1016/j.ajhg.2013.06.013 PMC3738825 23871722

[28] Moayyeri A, Hammond CJ, Hart DJ, Spector TD (2013) The UK Adult Twin Registry (TwinsUK Resource). Twin research and human genetics: the official journal of the International Society for Twin Studies 16: 144–9. 23088889 10.1017/thg.2012.89 PMC3927054

[29] Doughty MJ, Zaman ML (2000) Human corneal thickness and its impact on intraocular pressure measures: a review and meta-analysis approach. Survey of ophthalmology 44: 367–408. 10734239 10.1016/s0039-6257(00)00110-7

[30] Ojaimi E, Rose KA, Morgan IG, Smith W, Martin FJ, et al (2005) Distribution of ocular biometric parameters and refraction in a population-based study of Australian children. Investigative ophthalmology & visual science 46: 2748–54. 16043846 10.1167/iovs.04-1324

[31] Lee KE, Klein BE, Klein R, Quandt Z, Wong TY (2009) Association of age, stature, and education with ocular dimensions in an older white population. Archives of ophthalmology 127: 88–93. 19139346 10.1001/archophthalmol.2008.521 PMC2725427

[32] Rud E (1960) Megalocornea in a Danish gipsy family. Acta ophthalmologica 38: 606–17. 13744427 10.1111/j.1755-3768.1960.tb00227.x

[33] Cossette P, Liu L, Brisebois K, Dong H, Lortie A, et al (2002) Mutation of GABRA1 in an autosomal dominant form of juvenile myoclonic epilepsy. Nature genetics 31: 184–9. 11992121 10.1038/ng885

[34] Winnepenninckx B, Debacker K, Ramsay J, Smeets D, Smits A, et al (2007) CGG-repeat expansion in the DIP2B gene is associated with the fragile site FRA12A on chromosome 12q13.1. American journal of human genetics 80: 221–31. 17236128 10.1086/510800 PMC1785358

[35] Whibley AC, Plagnol V, Tarpey PS, Abidi F, Fullston T, et al (2010) Fine-scale survey of X chromosome copy number variants and indels underlying intellectual disability. American journal of human genetics 87: 173–88. 20655035 10.1016/j.ajhg.2010.06.017 PMC2917707

[36] Tarpey PS, Raymond FL, O'Meara S, Edkins S, Teague J, et al (2007) Mutations in CUL4B, which encodes a ubiquitin E3 ligase subunit, cause an X-linked mental retardation syndrome associated with aggressive outbursts, seizures, relative macrocephaly, central obesity, hypogonadism, pes cavus, and tremor. American journal of human genetics 80: 345–52. 17236139 10.1086/511134 PMC1785336

[37] Zou Y, Liu Q, Chen B, Zhang X, Guo C, et al (2007) Mutation in CUL4B, which encodes a member of cullin-RING ubiquitin ligase complex, causes X-linked mental retardation. American journal of human genetics 80: 561–6. 17273978 10.1086/512489 PMC1821105

[38] Badura-Stronka M, Jamsheer A, Materna-Kiryluk A, Sowinska A, Kiryluk K, et al (2010) A novel nonsense mutation in CUL4B gene in three brothers with X-linked mental retardation syndrome. Clinical genetics 77: 141–4. 20002452 10.1111/j.1399-0004.2009.01331.x

[39] Isidor B, Pichon O, Baron S, David A, Le Caignec C (2010) Deletion of the CUL4B gene in a boy with mental retardation, minor facial anomalies, short stature, hypogonadism, and ataxia. American journal of medical genetics Part A 152A: 175–80. 20014135 10.1002/ajmg.a.33152

[40] Dimasi DP, Burdon KP, Craig JE (2010) The genetics of central corneal thickness. The British journal of ophthalmology 94: 971–6. 19556215 10.1136/bjo.2009.162735

[41] Desmet FO, Hamroun D, Lalande M, Collod-Beroud G, Claustres M, et al (2009) Human Splicing Finder: an online bioinformatics tool to predict splicing signals. Nucleic acids research 37: e67. 19339519 10.1093/nar/gkp215 PMC2685110

[42] Reese MG, Eeckman FH, Kulp D, Haussler D (1997) Improved splice site detection in Genie. Journal of computational biology: a journal of computational molecular cell biology 4: 311–23. 9278062 10.1089/cmb.1997.4.311

[43] Hebsgaard SM, Korning PG, Tolstrup N, Engelbrecht J, Rouze P, et al (1996) Splice site prediction in Arabidopsis thaliana pre-mRNA by combining local and global sequence information. Nucleic acids research 24: 3439–52. 8811101 10.1093/nar/24.17.3439 PMC146109

[44] Piva F, Giulietti M, Nocchi L, Principato G (2009) SpliceAid: a database of experimental RNA target motifs bound by splicing proteins in humans. Bioinformatics 25: 1211–3. 19261717 10.1093/bioinformatics/btp124

[45] Wordinger RJ, Clark AF (2007) Bone morphogenetic proteins and their receptors in the eye. Exp Biol Med (Maywood) 232: 979–92. 17720944 10.3181/0510-MR-345

[46] Bakrania P, Efthymiou M, Klein JC, Salt A, Bunyan DJ, et al (2008) Mutations in BMP4 cause eye, brain, and digit developmental anomalies: overlap between the BMP4 and hedgehog signaling pathways. American journal of human genetics 82: 304–19. 18252212 10.1016/j.ajhg.2007.09.023 PMC2427285

[47] Rider CC, Mulloy B (2010) Bone morphogenetic protein and growth differentiation factor cytokine families and their protein antagonists. The Biochemical journal 429: 1–12. 20545624 10.1042/BJ20100305

[48] Abu A, Frydman M, Marek D, Pras E, Nir U, et al (2008) Deleterious mutations in the Zinc-Finger 469 gene cause brittle cornea syndrome. American journal of human genetics 82: 1217–22. 18452888 10.1016/j.ajhg.2008.04.001 PMC2427192

[49] Cameron JA (1993) Corneal abnormalities in Ehlers-Danlos syndrome type VI. Cornea 12: 54–9. 8458232 10.1097/00003226-199301000-00009

[50] Konig IR, Loley C, Erdmann J, Ziegler A (2014) How to include chromosome x in your genome-wide association study. Genetic epidemiology 38: 97–103. 24408308 10.1002/gepi.21782

