# Supplementary material for: Association of CHRDL1 Mutations and Variants with X-linked Megalocornea, Neuhäuser Syndrome and Central Corneal Thickness
Source: PLoS One. 2014 Aug 5;9(8):e104163. doi: 10.1371/journal.pone.0104163 (PMC4122416; doi:10.1371/journal.pone.0104163)
Supplement: File S1 — File includes Figures S1–S4 and Table S1. (DOC) [file pone.0104163.s001.doc]

**Supporting Information**


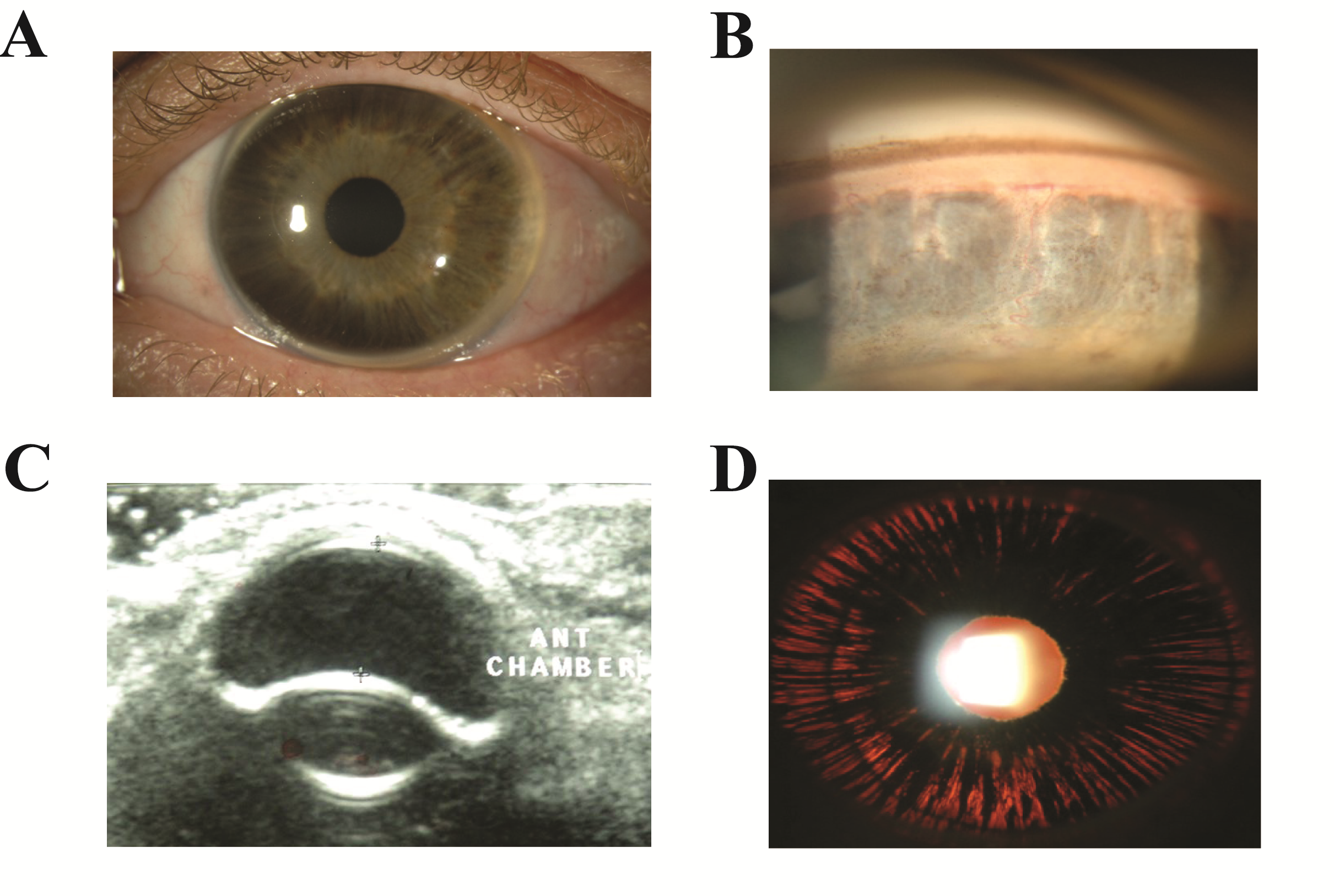


**Figure S1. Clinical images of Individual V:4 in Family A.**

**(A)** Left eye anterior segment, horizontal corneal diameter is 15 mm **(B)** Anterior chamber angle photograph showing marked posterior peripheral iris bowing and atrophic featureless peripheral iris with increased in typical pigmentation of the trabecular meshwork **(C)** B-mode ultrasound image of right eye demonstrating anterior chamber depth of 6.2 mm and posterior iris bowing in profile **(D)** Retroillumination of left eye peripheral showing marked peripheral iris transillumination that reveals the crystalline lens equator.


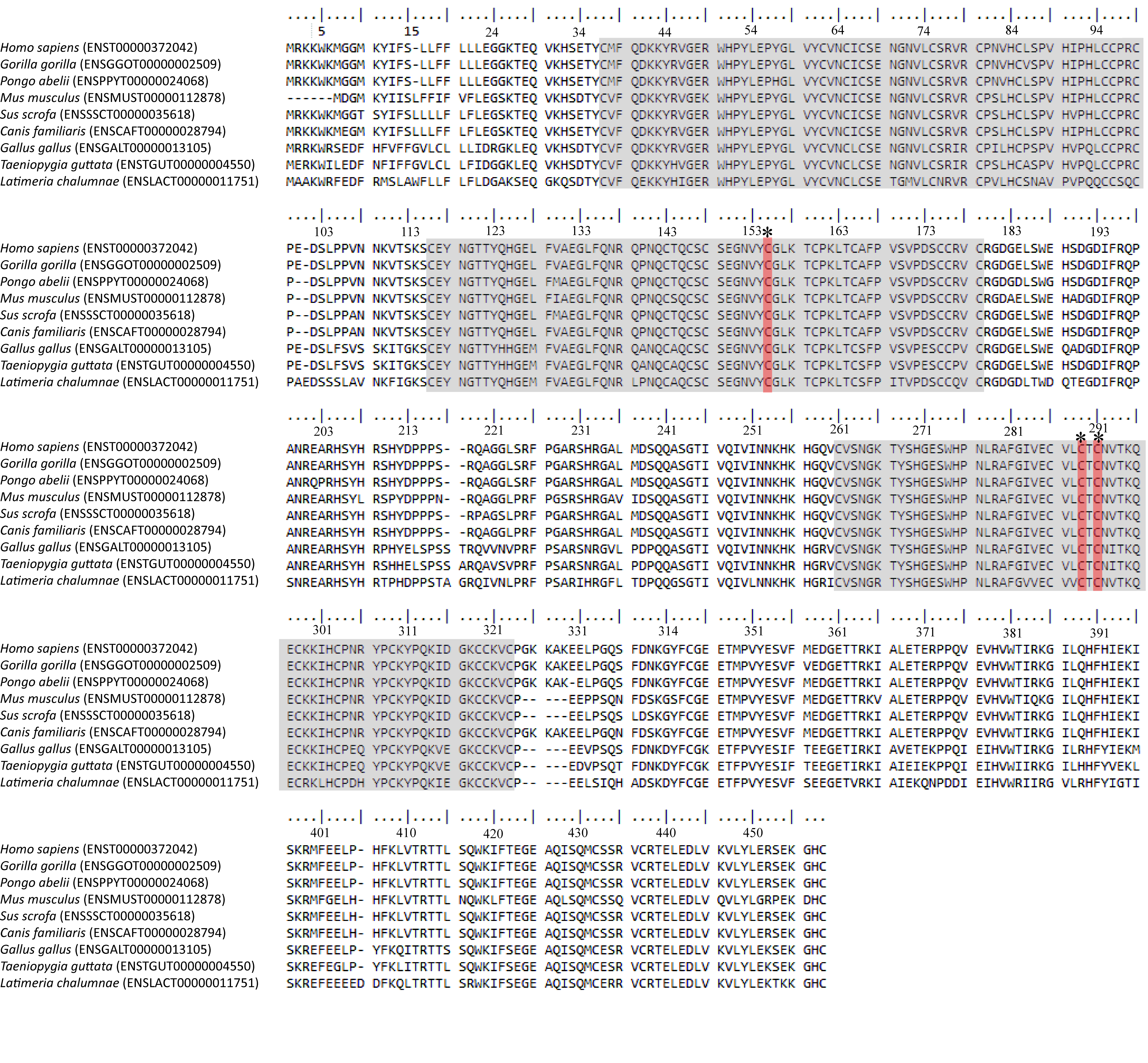


**Figure S2. Multiple sequence alignment of ventroptin orthologs**

Alignments performed with ClustalW. Shaded boxes denote three VWFC (von Willebrand factor, type C) domains. Asterisks indicate the conserved cysteine residues mutated in Family K c.464G>A; p.(Cys155Tyr), Family F c.865T>C; p.(Cys289Arg) and Family A c.872G>A; p.(Cys291Tyr). Accession numbers given represent transcript IDs from the Ensembl Genome Browser. The numbering of the amino acid residues is in accordance with human ventroptin sequence.


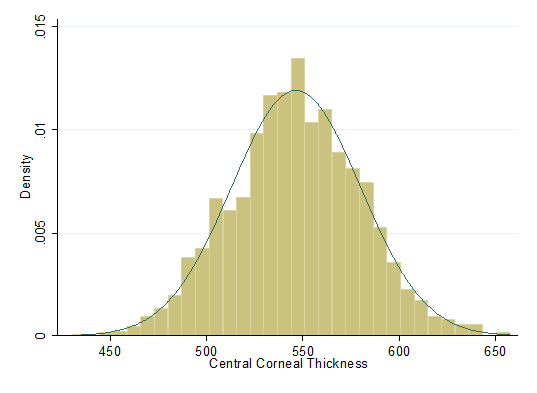


**Figure S3. Distribution of Central Corneal Thickness (CCT) observed in the TwinsUK cohort.**

The dark line represents the theoretically perfect normal distribution.


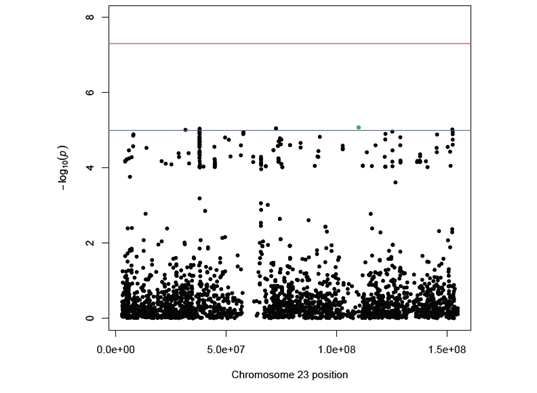


**Figure S4. Association of X chromosome SNPs with Central Corneal Thickness (CCT) in the TwinsUK cohort**

X chromosome association results (–log_10_ P values) are plotted for the TwinsUK cohort (N=1,957). The red line represents the formal GWAS significance threshold (that no SNP reached in this analysis). The most highly associated SNP, rs149956316, located within *CHRDL1* intron 6, is highlighted in green.

| **Table S1: Summary of rare variants identified in Individual III:4 (Family K) from WES data in genes associated with intellectual disability and/or hypotonia and/or seizures and/or epilepsy.** | | | | | | | | | | |
| --- | --- | --- | --- | --- | --- | --- | --- | --- | --- | --- |
| **Gene symbol** | **Nucleotide change** | **Protein change** | **Associated disease (MIM#)** | **Genomic coordinate** | **MAF ESP; X1000G** | **dbSNP** | **Polyphen 2 (human variation score 0-1)** | **SIFT (tolerance index 0-1)** | **Blosum 62 score**  **(-4 to 11)** | **Maternal Genotype** |
| *ADCK3* | Het  c.325C>G | p.(His109Asp) | (Rec) Primary coenzyme Q10 deficiency-4 (612016) | 1: 227,152,848 | 1/13,006; NA | [rs201479287](http://www.ncbi.nlm.nih.gov/projects/SNP/snp_ref.cgi?searchType=adhoc_search&type=rs&rs=201479287) | BNG (0.000) | T (0.61) | -1 | CC |
| *ERCC3* | Het  c.528C>T | p.(Phe176Phe) | (Rec) Trichothiodystrophy (601675)  (Rec) Xeroderma pigmentosum, group B (610651) | 2: 128,047,394 | NA; NA | rs114994654 | NA | NA | NA | CC |
| *UROC1* | Het c.1103 A>T | p.(Met368Lys) | (Rec) Urocanase deficiency (276880) | 3:126,219,580 | 1/13,006; NA | rs370883996 | PRD (0.988) | D (0.01) | -1 | TT |
| *ERCC8* | Het  c.152T>C | p.(Ile51Thr) | (Rec) Cockayne syndrome, type A (216400)  (Rec) UV-sensitive syndrome 2 (614621) | 5:60,224,712 | 1/13,000; NA | rs369140985 | PRD (0.988) | D (0.01) | -1 | TT |
| *GABRA1* | Het g.161326951A>C | NA (3’UTR) | (Dom) Childhood absence epilepsy, susceptibility to , 4 (611136) | 5:161,326,951 | NA; NA | NA | NA­­­­ | NA | NA | AA |
| *SEMA3E* | Het g.82995443A>G | NA (3’UTR) | (Chromosomal rearrangement) CHARGE syndrome (214800) | 7:82,995,443 | NA; NA | NA | NA | NA | NA | AG |
| *ERCC6* | Het g.50665953A>C | NA (3’UTR) | (Rec) Cerebrooculofacioskeletal syndrome 1 (214150)  (Rec) Cockayne syndrome, type B (133540)  (Rec) De Sanctis-Cacchione syndrome (278800)  (Rec) UV-sensitive syndrome 1 (600630) | 10:50,665,953 | NA; NA | NA | NA | NA | NA | AA |
| *DDB1* | Het  c.327C>T | p.(Thr109Thr) | Xeroderma pigmentosum, group E, subtype 2 | 11:61,091,490 | 20/13,004; 0.0005 | rs56096142 | NA | NA | NA | CT |
| *DIP2B* | Het c.3047 C>A | p.(Thr1016Asn) | (CGG expansion) FRA12A mental retardation (136630) | 12: 51,115,060 | NA; NA | NA | POS (0.582) | T(0.34) | 0 | CC |
| *CUL4B* | Hem  g.119709563delG | NA (5’UTR) | (X-linked recessive)  Mental retardation syndrome, X-linked 15 (Cabezas type) (300354) | X:119,709,563 | NA; NA | NA | NA | NA | NA | G- |
| WES was performed using the Illumina TruSeq exome enrichment (v3) and a HiSeq2000 sequencer (Illumina). Reads were aligned to the hg19 human reference sequence using Novoalign (Novocraft, www.novocraft.com) version 2.05. The ANNOVAR tool (OpenBioinformatics) was used to annotate SNPs and small insertions/deletions. Rare variants (MAF≤ 0.01) identified in genes associated with intellectual disability and/or hypotonia and/or seizures and/or epilepsy are shown above. WES analysis was also extended to include 5’UTR and 3’UTR variants, where covered. *In silico* analysis of rare variants identified is presented. Polyphen 2 appraises mutations quantitatively as benign (BNG), possibly damaging (POS) or probably damaging (PRD) based on the model’s false positive ratio. SIFT results are reported to be tolerant (T) if tolerance index is ≥0.05 or damaging (D) if tolerance index is <0.05. Blosum62 substitution matrix score positive numbers indicate a substitution more likely to be tolerated evolutionarily and negative numbers suggest the opposite. All variants presented were validated by Sanger sequencing using conventional methodologies. The unaffected mother (Individual II:2) and proband (III:4) in Family K share the same genotypes for rare variants found in *SEMA3E* and *DDB1*, paternal DNA was not available for testing. NA = not available; Rec = recessive; Dom = dominant. | | | | | | | | | | |
